# Niraparib induces hyperglycemia in ovarian cancer patients: a preliminary pilot study

**DOI:** 10.1007/s43440-025-00811-9

**Published:** 2025-12-10

**Authors:** Konrad Lewandowski, Joanna Stanisławiak-Rudowicz, Edyta Szałek, Anna Wolc, Agnieszka Karbownik

**Affiliations:** 1 Department of Clinical Pharmacy and Biopharmacy, Poznań University of Medical Sciences, Rokietnicka 3, Poznań, 60-806 Poland; 2 Doctoral School, Poznań University of Medical Sciences, Bukowska 70, Poznań, 60-812 Poland; 3 Poznań University Clinical Hospital, Szamarzewskiego 84/86, Poznań, 60-569 Poland; 4 Department of Animal Science, Iowa State University, 239E Kildee Hall, Ames, IA 50011 USA; 5 Dallas Center, Hy-Line International, 2583 240th Street, Dallas Center, IA 50063 USA

**Keywords:** Niraparib, Hyperglycemia, Adverse effect, Ovarian cancer, Body mass index (BMI)

## Abstract

**Background:**

Niraparib (Nir) is a poly(ADP-ribose) polymerase inhibitor (PARPi) used in the maintenance treatment of platinum-sensitive ovarian cancer (OC) patients, regardless of homologous recombination deficiency status. Being overweight or obese increases the risk of developing both OC and diabetes. Given this overlap, understanding the effect of Nir on glycemia is particularly important; however, it remains poorly understood.

**Methods:**

The study included 22 normoglycemic OC patients. Fasting glucose (FG) concentrations were measured before therapy and after the second, third, and fourth treatment cycle (a cycle is approximately 28 days). A linear mixed-effects model treating body mass index (BMI) as a continuous variable was applied for statistical calculations.

**Results:**

Impaired fasting glucose (IFG) (5.6–6.9 mmol/L) was observed in 55% of patients at some point during the study, and in 27% throughout its duration. A significant interaction between BMI and time was detected (*p* = 0.0412), with the influence of BMI on Nir-caused hyperglycemia increasing as the study progressed.

**Conclusions:**

Hyperglycemia appears to be an adverse effect of Nir. As there is some indication that BMI may influence this effect, glycemic control is recommended, particularly in patients with an elevated BMI.

**Supplementary Information:**

The online version contains supplementary material available at 10.1007/s43440-025-00811-9.

## Introduction

Ovarian malignancies are among the most lethal cancers in women worldwide, with 324,398 new cases in 2022 alone [1]. Up to 90% of ovarian cancers (OCs) are of epithelial origin [2, 3], and approximately 66% are diagnosed at an advanced stage, either FIGO (International Federation of Gynecological Oncology) stage III or IV [2]. Being overweight or obese is one of the factors that significantly increase the risk of ovarian cancer (OC), with obesity conferring a higher risk [4]. These factors also increase the risk of diabetes mellitus (DM) [5], which is itself a significant risk factor for OC in women and negatively affects survival and prognosis. The elevation of the insulin-like growth factor family in DM has been implicated as the cause of this relationship [6].

First-line treatment of OC typically involves cytoreductive surgery, followed by adjuvant platinum-based chemotherapy [2, 7]. Despite these interventions, recurrence occurs in about 80% of patients. Assessing the response to platinum compounds is crucial. Patients are generally categorized into two main groups: platinum-resistant patients who have progressed or recurred within 6 months, and platinum-sensitive patients who have achieved a partial or complete response to treatment and do not require chemotherapy for at least 6 months [8].

Susceptibility to platinum compounds determines subsequent therapeutic steps. In platinum-sensitive patients, a group of drugs called poly(ADP-ribose) polymerase inhibitors (PARPi) can be used, which significantly transformed the therapeutic prospects [9].

An important factor shaping clinical decisions in OC therapy is the genetic status, with homologous recombination deficiency (HRD) — a deficiency in the repair of double-strand DNA breaks — recognized as a key component. HRD is often caused by mutations in genes such as BRCA, which increase the risk of developing OC. HRD forces cells to rely on alternative DNA repair pathways, including the poly(ADP-ribose) polymerase (PARP) pathway. PARP is involved in DNA repair, primarily through the base excision repair pathway. The simultaneous inhibition of PARP activity in the presence of HRD leads to a phenomenon known as synthetic lethality, whereby the dysfunction of two distinct genes or pathways causes cell death, and the loss of only one is tolerable for the cell [10–12].

Niraparib (Nir), approved by the FDA (Food and Drug Administration) in 2017, is an example of a PARPi [9]. Nir has an absolute bioavailability of approximately 73%, with no apparent effect of food on its exposure, and is mainly metabolized by carboxylesterases. Protein binding is approximately 83%, the apparent volume of distribution is 1,311 L (for a 70 kg patient), and its half-life is approximately 2 days [13].

The results of the PRIMA, PRIME, and NOVA trials have led the American Society of Clinical Oncology (ASCO) to recommend the use of PARPi as maintenance therapy for both newly diagnosed and recurrent ovarian cancer (stage III-IV) in patients with a partial or complete response to platinum-based chemotherapy [9, 14]. In newly diagnosed patients, Nir is used as maintenance therapy at a starting dose of 200–300 mg orally once daily for up to 3 years. A dose of 300 mg is recommended for patients with a body weight ≥ 77 kg and a baseline platelet count ≥ 150 000/µL. If neither condition is met, the starting dose is 200 mg [13, 14].

However, it has been shown that the use of Nir as first-line therapy can be extended to all patients with platinum-sensitive advanced ovarian cancer, regardless of HRD status, without compromising clinical benefit, which is not the case for all PARPi, making Nir a more universally applicable option [9].

Treatment with Nir is not without side effects. The most common are hematological (e.g., thrombocytopenia, anemia, neutropenia) and gastrointestinal (e.g., nausea, vomiting, and constipation) toxicities. According to the Common Terminology Criteria for Adverse Events (CTCAE, version 5.0 [15]), Grade 3 adverse events most frequently include fatigue, anemia, thrombocytopenia, and neutropenia. Moreover, the most serious adverse events (grade 3 or higher) typically occurred within the first 3 months of treatment.

Clinical trials (NOVA, PRIMA, NORA) have shown that approximately 60–70% of patients required dose modifications for this reason, with thrombocytopenia being the most common reason for dose reduction. This led to adjustments in initial dosing recommendations based on patients’ platelet counts and body weight without compromising treatment efficacy as measured by progression-free survival (PFS). These adjustments reduced the discontinuation rate and incidence of thrombocytopenia by almost 25% points. No hyperglycemia was reported [16]. A recent meta-analysis suggests that Nir may induce hyperglycemia [17]. However, not all of the included studies focused on OC, and many of the earlier studies did not report this adverse effect [16, 18]. Furthermore, whether body mass index (BMI) influences this side effect has not yet been investigated.

Moreover, for another PARPi, olaparib, a significant increase in patients’ glycaemia was observed. Differences in the occurrence of hyperglycemia depended on patients’ BMI and treatment duration. In patients with a lower BMI, significant hyperglycemia occurred earlier than in obese patients [7].

Therefore, due to the lack of data, our aim is to conduct a pilot study to determine whether Nir affects glycaemia in patients with epithelial OC, and to determine whether this effect is influenced by BMI.

## Materials and methods

### Study subjects

**Fig. 1:**
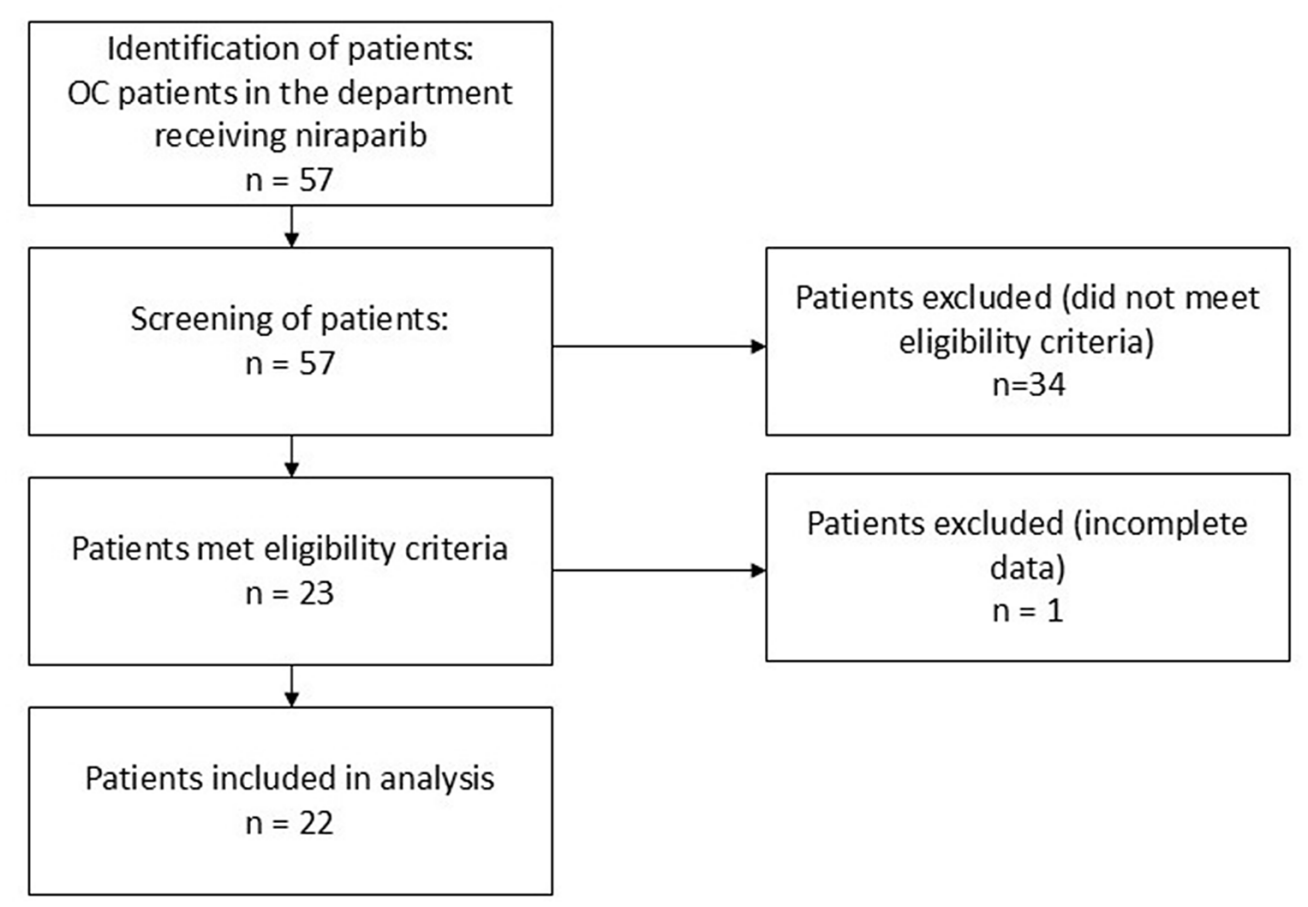
PRISMA flowchart showing the selection of OC patients receiving Nir. *OC* – ovarian cancer; *Nir* – niraparib. The open-label study was conducted between January 2022 and November 2024 at the Department of Gynecological Oncology and the Department of Clinical Pharmacy and Biopharmacy of Poznań University of Medical Sciences, Poland

The open-label retrospective study was conducted at the Department of Gynecological Oncology and the Department of Clinical Pharmacy and Biopharmacy of Poznań University of Medical Sciences, Poland. The study protocol was approved by the Bioethics Committee at Poznań University of Medical Sciences, Poland (protocol no. 733/24). All procedures conducted in this study—including the planning phase, all research procedures, and the plan for dissemination of the results obtained, were prepared in accordance with the Declaration of Helsinki [19], the local regulations on patient confidentiality (Dz. U. 2009 Nr 52 poz. 417), and the local implementation of the General Data Protection Regulation (GDPR), applicable in the European Member states since 2018 [20]. To ensure the privacy and confidentiality of data, all sensitive information was removed from the database prior to analysis. Subjects included OC patients who received Nir at the Department of Gynecological Oncology between January 2022 and April 2025. The inclusion criteria for this study were: age >18 years, no history of allergy to Nir, normoglycemia (≤ 5.5 mmol/L) before Nir treatment, and at least 4 months of Nir treatment. Participants were excluded from the study if they met any of the following criteria: allergy to Nir, age < 18 years, diabetes, or a pre-diabetic state (Impaired fasting glucose (IFG) was the most common criterion for patient exclusion affecting 34 patients. Some patients may have exhibited transient abnormal glycaemia due to glucocorticosteroids administered as part of premedication with taxanes). Informed consent was obtained from all the subjects enrolled in the study. The selection of patients is shown in Fig. 1.

### Study design

OC patients (*n* = 22) received Zejula ^®^ in capsules (January 2022 – November 2024) or tablets (from December 2024). Nir was administered once daily at a dose of 300 mg, 200 mg, or 100 mg. The initial dose was chosen based on body weight and platelet count. Any subsequent reduction was determined in accordance with the Summary of Product Characteristics [13]. Fasting glycaemia levels were verified before Nir treatment (time point 0 - T0), after the second (time point 1 - T1), third (time point 2 - T2), and fourth treatment cycles (time point 3 - T3). The date of the end of the cycle was defined as the time when the next pack of medication was dispensed to the patient. If the patient did not stop taking the medication during the course, it took approximately 4 weeks. Cycle 1 was omitted due to the frequent need for periodic breaks in taking the medicine and for dose reductions in the early phase due to hematological side effects. The effect of Nir on glycaemia was assessed using fasting plasma glucose (FPG). The FPG criterion for diabetes is ≥ 7.0 mmol/L. A value between 5.6 and 6.9 mmol/L is known as IFG and indicates a pre-diabetic state [21]. The severity of hyperglycemia was graded according to the CTCAE [15]. The doses patients received are presented in Table 1.

**Table 1:** Doses of Nir administered to patients at subsequent time points throughout a course of treatment

Dose [mg]	Number of patients (*N* = 22)			
	T0	T1	T2	T3
300	2	1	0	0
200	20	12	10	10
100	0	9	12	12

*Nir* – niraparib; *T0*, *T1*, *T2*, *T3* – time points throughout the course of treatment (*T0* - time point 0, before Nir treatment; *T1* - time point 1, after the second cycle of Nir treatment, *T2* - time point 2, after the third cycle of Nir treatment; *T3* - time point 3, after the fourth cycle of Nir treatment. The end of the cycle was defined as the time when the next pack of medication was dispensed to the patient), *N* – total sample size

The open-label study was conducted between January 2022 and November 2024 at the Department of Gynecological Oncology and the Department of Clinical Pharmacy and Biopharmacy of Poznań University of Medical Sciences, Poland

At T0, 9% of patients were on a 300 mg dose, compared to 0% at T2, while 91% were on a 200 mg dose, compared to 45% at T2. At T2 and T3 55% were on a 100 mg dose. Across T1, T2, and T3, 4 patients (18%) underwent a dose reduction; all other dose reductions occurred between the start of therapy and T1.

### Statistical analysis

Statistical analyses were conducted using the R software (version 4.4.3). A linear mixed-effects model treating BMI as a continuous variable was fitted using the *lmer* function from the *lmerTest* package (version 3.1.3). The applied model was: lmer(glycaemia ~ time * BMI (continuous) + dose + (1|patient’s ID),* data = database).* Normality was assessed using the Shapiro–Wilk test. The dose was computed by assigning the dose value from the previous time point to the glycaemia measured at the current time point. For the baseline measurement (T0), a dose of 0 was assumed. Post hoc comparisons were performed using the *emmeans* package (version 1.11.1), with Holm adjustment at representative BMI values: at the sample mean (BMI = 25.0 kg/m^2^), and at ± one standard deviation (BMI = 21.8 kg/m^2^ and BMI = 28.1 kg/m^2^). A significance level of α = 0.05 was assumed. In addition, the effect size was examined. The following effect size measures were used for the tests: linear mixed-effects model - partial eta-squared (η²_p_) calculated using the *effectsize* package (version 1.0.0), based on Type III sum of squares; post-hoc pairwise comparisons with Holm correction for linear mixed-effects model - Cohen’s d_z_ (1) [22]:

1 $$\:{d}_{z}=\frac{t}{\sqrt{n}}$$

t – t-statistic

n – total number of participants

## Results

### Patient characteristic

The mean age of patients was 64 years, with a standard deviation of 15 years. The youngest subject was 28 years old, while the oldest was 85. The same number of patients had both normal BMI and overweight (*n* = 10). The mean BMI was 24.97 kg/m². Patients were at various stages of the disease. The most common stage was FIGO IIIC (*n* = 12). The majority of patients (*n* = 19) were diagnosed with serous ovarian carcinoma. All tumors were classified as high-grade. The two most prevalent comorbidities in the study group were arterial hypertension (*n* = 9) and cardiac arrhythmia (*n* = 4). The most commonly used concomitant medications were consequently ACE inhibitors or sartans (*n* = 9), beta-blockers (*n* = 7), statins (*n* = 5), and dihydropyridine calcium channel blockers (*n* = 4). The characteristics of the patients are shown in Table 2.

**Table 2:** Patient characteristics

Variable		Value
**Age**		
**Mean [min; max]** **Median [Q1; Q3]**		64 [28; 85] 68 [50; 74]
**BMI** [*n* **(%)]**		
18.5–24.9 kg/m²		10 (45)
25.0–29.9 kg/m²		10 (45)
≥ 30 kg/m²		2 (9)
**FIGO Stage** [*n* **(%)]**		
III A		1 (4)
III B		3 (14)
III C		12 (54)
IV A		3 (14)
IV B		3 (14)
**Histological type** [*n* **(%)]**		
serous		19 (86)
serous-endometrial		1 (4)
serous–mucinous		1 (4)
adenocarcinoma not otherwise specified		1 (4)
**Comorbidity** [*n* **(%)]**		
arterial hypertension		9 (41)
cardiac arrhythmia		4 (18)
hypothyroidism		3 (14)
asthma		2 (9)
hypercholesterolemia		2 (9)
depression		1 (4)
Parkinson’s disease		1 (4)
diverticular disease of the large intestine		1 (4)
cervical discopathy		1 (4)
rheumatoid arthritis		1 (4)
**Concomitant medications** [*n* **(%)]**		
ACE-I/ sartans		9 (41)
beta-blockers		7 (32)
statins		5 (23)
calcium channel blockers of the dihydropyridine class		4 (18)
levothyroxine		3 (14)
vinpocetine		2 (9)
SSRIs		2 (9)
antiarrhythmic drugs		1 (4)
H1-blockers		1 (4)
mebeverine		1 (4)
levodopa		1 (4)
diuretic		1 (4)
allopurinol		1 (4)
ezetimibe		1 (4)
acetylsalicylic		1 (4)
enoxaparin		1 (4)
**Biochemical and hematological parameters** **Mean ± SD** **Median [Q1; Q3]**		
WBC		5.69 ± 2.02 5.40 [4.25; 6.84]
NEUT		3.24 ± 1.66 2.90 [2.15; 3.53]
RBC		3.64 ± 0.37 3.55 [3.49; 3.90]
HGB		7.46 ± 0.64 7.50 [6.98; 7.88]
HCT		0.36 ± 0.03 0.37 [0.34; 0.38]
PLT		246.91 ± 120.97 214 [184.25; 259.25]
ALAT		23.27 ± 10.08 20.50 [16.25; 28.00]
ASPAT		24.59 ± 5.23 25.00 [20.00; 27.50]
BIL		7.20 ± 3.72 7.40 [3.85; 10.00] *n* = 20
CREAT		69.91 ± 17.16 67.5 [57; 77.75]

*Mean* – arithmetic mean; *SD* – standard deviation; *Q1-Q3 –* Quartile; *min* – minimum; *max –* maximum; *BMI* – Body Mass Index; *[n (%)]* – number of patients and percentage of the whole group; *FIGO* – International Federation of Gynaecological Oncology; *WBC* – white blood cells; *NEUT* – neutrophils; *RBC* – red blood cells; *HBG –* hemoglobin; *HCT –* hematocrit; *PLT* – platelet count; *ALT* – alanine transaminase; *AST* – aspartate transaminase; *BIL* – bilirubin; *CREAT* – creatinine

The open-label study was conducted between January 2022 and November 2024 at the Department of Gynecological Oncology and the Department of Clinical Pharmacy and Biopharmacy of Poznań University of Medical Sciences, Poland

### Glycaemia levels

The mean blood glucose level for the entire study group at the onset of treatment was 5.18 ± 0.26 mmol/L. The highest recorded glycaemia was 7.3 mmol/L at T3 in one of the obese patients. The same patient achieved the greatest overall change from 4.7 mmol/L at baseline to 7.3 mmol/L at T3. This is also the only patient to exceed 7 mmol/L at any of the time points. IFG (5.6–6.9 mmol/L) was observed in 12 (55%) patients at any one time point and in 6 (27%) at all three time points. According to the CTCAE [15], grade 1 hyperglycemia was recorded in 11 patients (50%), and grade 2 in one patient (5%). The glycemic values at specific time points are presented in Table 3.

**Table 3:** Glycemic levels before and after the second, third, and fourth cycle of Nir treatment in OC patients

Cycle of Nir treatment	Glucose level [mmol/L] *N* = 22
	Mean ± SD Median [Q1, Q3]
T0	5.18 ± 0.26 5.30 [4.93; 5.40]
T1	5.55 ± 0.43 5.60 [5.25; 5.78]
T2	5.54 ± 0.49 5.40 [5.20; 5.98]
T3	5.54 ± 0.65 5.4 [5.12; 5.68]

*Nir* – niraparib; *OC* – ovarian cancer; *T0*, *T1*, *T2*, *T3* – time points throughout the course of treatment (*T0* - time point 0, before Nir treatment; *T1* - time point 1, after the second cycle of Nir treatment, *T2* - time point 2, after the third cycle of Nir treatment; *T3* - time point 3, after the fourth cycle of Nir treatment. The end of the cycle was defined as the time when the next pack of medication was dispensed to the patient), *Mean -* arithmetic mean, *SD -* standard deviation, *Q1-Q3 -* Quartile, *N* – total sample size

The open-label study was conducted between January 2022 and November 2024 at the Department of Gynecological Oncology and the Department of Clinical Pharmacy and Biopharmacy of Poznań University of Medical Sciences, Poland

### The effect of niraparib on patients’ glycemic levels

**Fig. 2:**
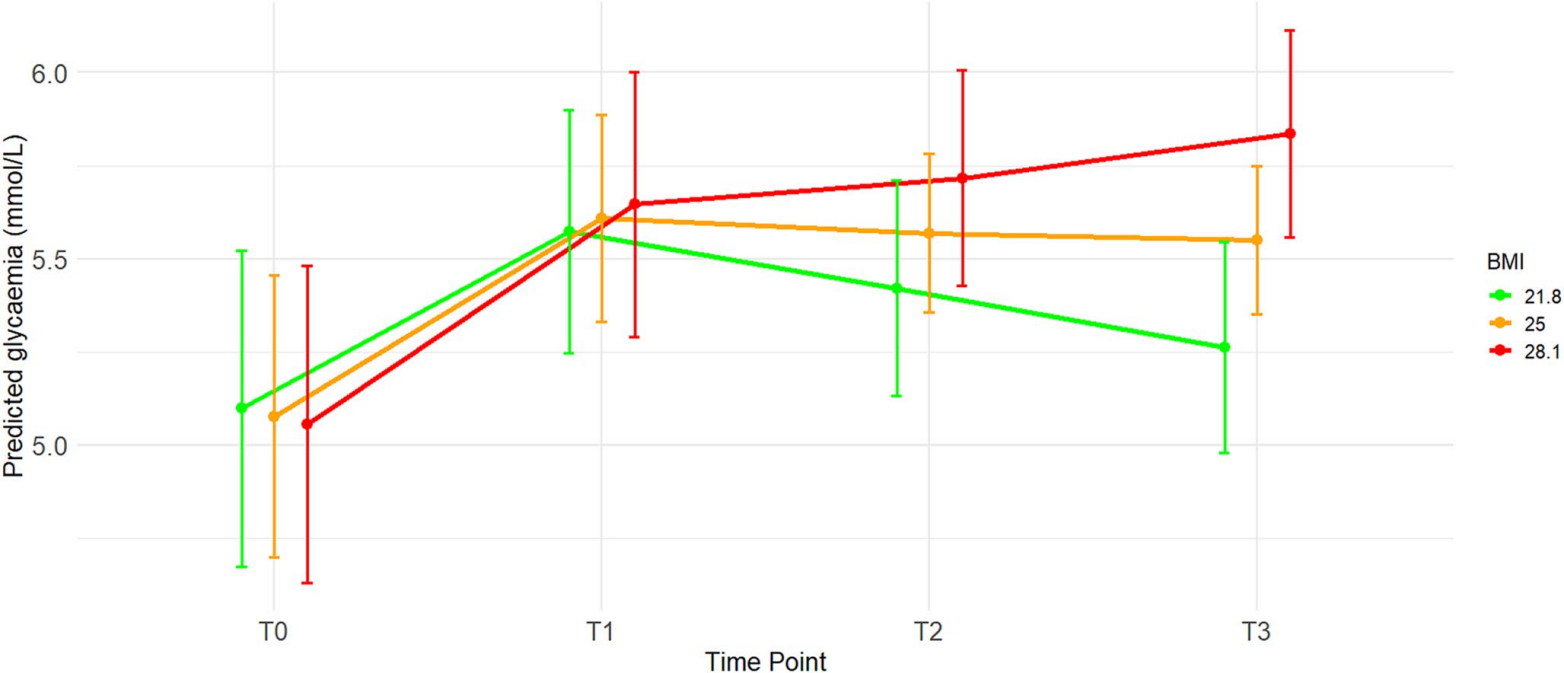
Predicted glycaemia across time points (T0–T3) at representative BMI values: the sample mean (BMI = 25.0 kg/m^2)^ and ± one standard deviation (BMI = 21.8 kg/m^2^ and BMI = 28.1 kg/m^2^) based on the linear mixed-effects model [lmer(glycaemia ~ time * BMI (continuous) + dose + (1|patient’s ID),* data = database)]. Nir – niraparib; T0, T1, T2, T3 – time points throughout the course of treatment (T0 - time point 0, before Nir treatment; T1 - time point 1, after the second cycle of Nir treatment, T2 - time point 2, after the third cycle of Nir treatment; T3 - time point 3, after the fourth cycle of Nir treatment. The end of the cycle was defined as the time when the next pack of medication was dispensed to the patient), BMI - Body Mass Index. *The open-label study was conducted between January 2022 and November 2024 at the Department of Gynecological Oncology and the Department of Clinical Pharmacy and Biopharmacy of Poznań University of Medical Sciences, Poland. Vertical bars represent 95% confidence intervals of the mean

A linear mixed-effects model revealed a significant interaction between time and BMI, as presented in Table 4. None of the other factors in the model was significant. Changes in glycemia over time depend on BMI values: the higher the BMI, the greater the increase in glycemia. The patient’s BMI value influences Nir-induced hyperglycemia. Figure 2 shows the glycemic values predicted by the model for the selected BMI values at specific time points. Post hoc comparisons (Table S1) showed that glycemic changes for people with a low BMI (21.8 kg/m^2^) and medium BMI (25.0 kg/m^2^) were not significant for the adopted model, whereas changes for people with a high BMI (28.1 kg/m^2^) were significant only between time points T0 and T3. Effect sizes (η²_p_) indicated that the interaction between duration and BMI explained around 13% of the variance in glucose levels.

**Table 4:** The impact of BMI, time, interaction between BMI and time, dose on glycaemia levels in patients undergoing therapy with Nir based on a linear mixed-effect model [lmer(glycaemia ~ time * BMI (continuous) + dose + (1|patient’s ID),* data = database)]*

Effect	df	F - statistic	η²_*p*_	*p*-value
BMI	1, 19.60	2.54	0.115	0.1269
time	3, 59.97	2.18	0.098	0.0995
BMI x time	3, 59.52	2.92	0.128	**0.0412**
dose	1, 79.00	0.43	0.005	0.5115

*BMI* - Body Mass Index; *Nir* – niraparib; *BMI x time* – interaction between BMI and time; *df*- degrees of freedom, η²_p_ partial eta-squared (effect size)

The open-label study was conducted between January 2022 and November 2024 at the Department of Gynecological Oncology and the Department of Clinical Pharmacy and Biopharmacy of Poznań University of Medical Sciences, Poland

## Discussion

We have developed a linear mixed-effects model to investigate the effect of Nir on glycaemia and its possible relationship with BMI values. Regardless of the model, we found that Nir affects patients’ glycemic levels. In more than half of the patients (55%), IFG (5.6–6.9 mmol/L) was observed during the study, and in more than one-quarter of the patients (27%), it persisted throughout the study duration.

Some of the patients in the study group (*N* = 20) had previously undergone a test to assess the short-term effect of Nir on glycaemia after administration of the drug. Glycemic values were compared before and after three months of Nir therapy using a paired t-test. Significant results were obtained (*p* = 0.0270; effect size: grm – 0.7397) [23]. However, we considered it necessary to include the BMI value of the patients in the analysis. BMI is a strong indicator of the occurrence of diabetes, even stronger than genetic risk [24]. Nir and BMI would therefore be two independent factors in the development of hyperglycemia. This enabled us to investigate whether there might be any relationship between these factors and whether BMI could influence the effects of Nir on glycaemia. This is the second PARPi drug for which we have observed an effect on carbohydrate metabolism. For olaparib, we have shown that, similarly to these results, about 50% of patients experience hyperglycemia [7]. The olaparib study, in contrast to this model, noted the influence of BMI (divided into three groups: normal BMI, overweight, and obese) on the onset of hyperglycemia in patients during treatment. This effect appeared earlier in the group of patients with lower BMI than in the group of overweight and obese patients [7].

The linear mixed-effects model (Table 4) showed slightly different results. No significant effect of time or BMI was found. However, the interaction between time and BMI as a continuous variable had the largest significant impact on the model, indicating that glycemic changes at specific time points were influenced by BMI values. A patient with a higher BMI may experience an upward influence on Nir-induced hyperglycemia. This may suggest that two independent factors of hyperglycemia, BMI and Nir, may show a relationship in clinical practice. For this model, we compared time points for the selected representative BMI values of our patient group (i.e., the sample mean of 25.0 kg/m² and the upper and lower limits of one standard deviation, i.e., 21.8 kg/m² and 28.1 kg/m², respectively) (see Fig. 2 and Table S1). We demonstrated that the influence of low and intermediate BMI values on Nir-induced glycaemia was not significant. In contrast, for a high BMI, there was a continuous increase in glycaemia at successive time points. It should be noted that this model assumes a linear, monotonic relationship between BMI and glycaemia over time. This implies a constant increase (or decrease) in glycaemia with increasing BMI, without accounting for potential non-linear effects, such as stabilization.

The EVERMET study yielded noteworthy results. In patients with advanced breast cancer treated with a combination of exemestane and everolimus, an mTOR inhibitor, it has been demonstrated that patients who are normoglycemic at baseline and experience on-treatment diabetes have a reduced PFS in comparison to patients who were already hyperglycemic at baseline (mPFS 6.34 vs. 10.32 months; HR 1.76; 95% CI 1.15–2.69; *p* = 0.008). It is hypothesized that this is due to the reactivation of the IR/PI3K/AKT/mTORC1 pathway by diabetes, as hyperglycemia and hyperinsulinemia are class effects of PI3K/AKT/mTORC1 axis inhibitors [25]. The question remains whether a similar situation can occur with Nir. Importantly, it appears that mTORs block the physiological pathway responsible for carbohydrate metabolism, and such a mechanism for causing hyperglycemia is not possible for Nir. It would be worth investigating whether on-treatment hyperglycemia is associated with a decreased PFS and whether terminating Nir therapy affects glycaemia.

Our results have been confirmed by a meta-analysis conducted by Fu et al. [17] in which Nir demonstrated an increased risk of any-grade hyperglycemia (OR = 2.15, 95% CI 1.28–3.62) in patients with solid tumors. It is thought that the mechanism may be related to the inhibition of dopamine and norepinephrine transporters, thereby impairing their cellular uptake [17]. Moreover, no significant effect on blood glucose has been demonstrated for olaparib, which contradicts our results from the previous publication [7]. In addition, rucaparib, another PARPi, is likely to have a mitigating effect on hyperglycemia [17]. These results suggest that Nir-induced hyperglycemia is not a class effect and does not affect all PARPi. This aligns with the findings of Sandhu et al. [26], where, despite a similar mode of action, each of the PARPi may exhibit characteristic adverse effects.

In general, DM negatively impacts survival and prognosis for OC patients [6]. It is noteworthy that metformin remains one of the first-line drugs for hyperglycemia [13]. Despite its relatively low potential for interaction, the Summary of Product Characteristics states that Nir is an inhibitor of MATE1 and MATE2 and is also a weak inhibitor of organic cation transporter 1 (OCT1); therefore, caution is advised when Nir is combined with metformin [13]. The combination of metformin and Nir could lead to an increased plasma metformin concentration and a higher risk of metformin adverse effects, including lactic acidosis [13, 27]. Moreover, metformin has anti-tumor properties through multiple mechanisms, including reduced neovascularization, inhibition of several key OC tyrosine kinase receptors, such as HER4, epidermal growth factor, and platelet-derived growth factor receptor, or inhibition of the PI3K/AKT/mTOR pathway [6]. Co-administration of Nir and metformin could prove beneficial, but attention should be paid to potential pharmacokinetic interactions. In addition, an in vitro study by Shang et al. found that the combination of metformin and telmisartan inhibited PARP1 activity via AMP-activated protein kinase (AMPK) phosphorylation, which had a positive effect on alleviating endothelial dysfunction [28]. This seems particularly beneficial given that hypertension is one of the adverse effects of Nir [13].

This pilot study has several limitations. Firstly, it was retrospective and included a small number of patients. The study was characterized by low statistical power, necessitating both internal and external validation. Furthermore, fasting blood glucose was the only parameter used to evaluate the carbohydrate metabolism of these patients. Hemoglobin A1c, one of the primary tools for assessing glycemic status in clinical practice, strongly linked to diabetes complications, was not assessed [29]. The group also had considerable comorbidities and medication, which could have influenced the results. Moreover, patients experienced treatment interruptions due to adverse, particularly hematological, side effects. Temporary discontinuation of therapy and/or dose reduction affected 15 patients (68%). The most prevalent cause of dose reduction was a decreased platelet count, accounting for 79% of cases. According to the CTCAE [15], 36%, 45%, and 9% of these cases were characterized as grade 2, 3, and 4, respectively.

## Conclusions

Hyperglycemia was recorded in more than 50% of patients during the study; therefore, it is recommended that patients’ blood glucose levels be monitored and glycated hemoglobin be routinely tested to prevent diabetic complications. Moreover, continuous glucose monitoring should be considered in the early stages of therapy to obtain a complete glycemic profile of patients. There is some indication that Nir-induced hyperglycemia may be influenced by BMI. Further research is required to understand the mechanism by which Nir induces hyperglycemia and to investigate whether this may have an impact on patients’ PFS or overall survival, and whether terminating Nir therapy affects glycaemia. Overall, developing recommendations for glycemic monitoring in patients treated with PARP inhibitors could be a valuable endeavor in future studies.

## Supplementary Information

Below is the link to the electronic supplementary material.

Supplementary Material 1

## Data Availability

The datasets generated during and/or analyzed during the current study are available from the corresponding author upon reasonable request.

## Abbreviations

AMPK: AMP-activated protein kinase
ASCO: American Society of Clinical Oncology
BMI: Body Mass Index
CTCAE: Common Terminology Criteria for Adverse Events
DM: Diabetes mellitus
FDA: Food and Drug Administration
FG: Fasting glucose
FPG: Fasting plasma glucose
FIGO: International Federation of Gynaecological Oncology
GDPR: General Data Protection Regulation
HRD: Homologous recombination deficiency
IFG: Impaired fasting glucose
mTORi: mTOR inhibitors
mTORC1: mTOR complex 1
NCN: National Science Centre
Nir: Niraparib
OC: Ovarian cancer
OCT1: Organic cation transporter 1
PARP: Poly(ADP-ribose) polymerase
PARPi: Poly(ADP-ribose) polymerase inhibitors
PFS: Progression-free survival
TKI: Tyrosine kinase inhibitors
T0: Time point 0
T1: Time point 1
T2: Time point 2
T3: Time point 3
