# Supplementary material for: Niraparib induces hyperglycemia in ovarian cancer patients: a preliminary pilot study
Source: Pharmacol Rep. 2025 Dec 10;78(2):597–606. doi: 10.1007/s43440-025-00811-9 (PMC12975802; doi:10.1007/s43440-025-00811-9)
Supplement: Supplementary file 1 — Supplementary Material 1 [file 43440_2025_811_MOESM1_ESM.docx]

**Table S1** Pairwise comparisons between time points for glycaemia with Holm-corrected *p*-values at representative BMI values: the sample mean (BMI = 25.0 kg/m^2)^ and ± one standard deviation (BMI = 21.8 kg/m^2^ and BMI = 28.1 kg/m^2^) for linear mixed-effects model [*lmer(glycaemia ~ time * BMI (continuous) + dose + (1|patient’s ID), data = database)]*

|  | Mean difference | SE | df | t-statistic | Effect size | p-value |
| --- | --- | --- | --- | --- | --- | --- |
| BMI = 21.8 kg/m^2^ | | | | | | |
| T0-T1 | -0.4751 | 0.295 | 76.9 | -1.612 | 0.3437 | 0.5550 |
| T0-T2 | -0.3227 | 0.258 | 75.5 | -1.251 | 0.2667 | 0.8585 |
| T0-T3 | -0.1647 | 0.249 | 75.0 | -0.662 | 0.1411 | 0.9526 |
| T1-T2 | 0.1525 | 0.163 | 61.2 | 0.938 | 0.2000 | 0.9526 |
| T1-T3 | 0.3104 | 0.166 | 62.3 | 1.870 | 0.3987 | 0.3971 |
| T2-T3 | 0.1579 | 0.157 | 59.2 | 1.008 | 0.2150 | 0.9526 |
| BMI = 25.0 kg/m^2^ | | | | | | |
| T0-T1 | -0.5325 | 0.285 | 78.4 | -1.870 | 0.3987 | 0.2609 |
| T0-T2 | -0.4912 | 0.233 | 77.7 | -2.107 | 0.4492 | 0.1919 |
| T0-T3 | -0.4720 | 0.213 | 77.1 | -2.213 | 0.4718 | 0.1793 |
| T1-T2 | 0.0413 | 0.124 | 65.1 | 0.332 | 0.0708 | 1.0000 |
| T1-T3 | 0.605 | 0.136 | 68.8 | 0.444 | 0.0947 | 1.0000 |
| T2-T3 | 0.0192 | 0.113 | 60.2 | 0.171 | 0.3646 | 1.0000 |
| BMI = 28.1 kg/m^2^ | | | | | | |
| T0-T1 | -0.5899 | 0.317 | 77.4 | -1.863 | 0.3972 | 0.2649 |
| T0-T2 | -0.6598 | 0.259 | 75.5 | -2.552 | 0.5441 | 0.0636 |
| T0-T3 | -0.7793 | 0.232 | 73.7 | -3.359 | 0.7161 | **0.0075** |
| T1-T2 | -0.0700 | 0.171 | 63.8 | -0.409 | 0.0872 | 0.9485 |
| T1-T3 | -0.1894 | 0.188 | 67.8 | -1.010 | 0.2153 | 0.9485 |
| T2-T3 | -0.1195 | 0.160 | 60.4 | -0.747 | 0.1593 | 0.9485 |

*Nir* – niraparib; *T0*, *T1*, *T2*, *T3* – time points throughout the course of treatment (*T0* - time point 0, before Nir treatment; *T1* - time point 1, after the second cycle of Nir treatment, *T2* - time point 2, after the third cycle of Nir treatment; *T3* - time point 3, after the fourth cycle of Nir treatment. The end of the cycle was defined as the time when the next pack of medication was dispensed to the patient), *BMI* - Body Mass Index; *SE* – standard error; *df* – degrees of freedom

The open-label study was conducted between January 2022 and November 2024 at the Department of Gynecological Oncology and the Department of Clinical Pharmacy and Biopharmacy of Poznań University of Medical Sciences, Poland
